# Evaluation the effects of red yeast rice in combination with statin on lipid profile and inflammatory indices; a randomized clinical trial

**DOI:** 10.1186/s40795-022-00639-z

**Published:** 2022-11-25

**Authors:** Ali Tavan, Saam Noroozi, Bardia Zamiri, Reza Gholchin Vafa, Mohammadhossein Rahmani, Mohammadjavad Mehdizadeh Parizi, Amin Ahmadi, Reza Heydarzade, Mohammad Montaseri, Seyed Ali Hosseini, Javad Kojuri

**Affiliations:** 1 Professor Kojuri Cardiology Clinic, Shiraz University of Medical Sciences, Niayesh Medical Complex, Niayesh St, Shiraz, Iran; 2 Fasa University of Medical Sciences, Fasa, Iran; 3 Cardiology Department, Shiraz University of Medical Sciences, Shiraz, Iran; 4 Clinical Education Research Center, Shiraz University of Medical Sciences, Shiraz, Iran

**Keywords:** Hyperlipidemias, Hydroxymethylglutaryl-CoA reductase inhibitors, Red yeast rice, Cardiovascular diseases

## Abstract

**Background:**

Dyslipidemia is a prominent cause of cardiovascular disease as it leads to inflammation and plaque deposition within arteries. Treatment includes lifestyle modifications and lipid-lowering medications. We aimed to assess the therapeutic effects of red yeast rice (RYR) alongside statin therapy.

**Methods:**

This triple-blind randomized clinical trial involved 92 dyslipidemia patients and was performed in 2019. Standard laboratory tests were used to assess the serum LDL cholesterol (LDL-C), HDL cholesterol (HDL-C), total cholesterol, triglyceride (TG), and high sensitivity C-reactive protein (hs-CRP) levels. Subsequently, patients randomly received one daily RYR or placebo tablet for 1 month beside routine single statin therapy. Subsequently, blood tests were repeated and compared against the baseline. Liver function tests were also requested.

**Results:**

Total cholesterol significantly (*P* = 0.019) decreased in the treatment group (− 10.2 mg/dL) compared with the placebo group (− 1.3 mg/dL). HDL cholesterol decreased by 2.19 mg/dL in the treatment group but increased by 0.53 mg/dL in the treatment group (*P* = 0.083). LDL cholesterol declined in both placebo (− 5.09) and treatment (− 0.73) groups (*P* = 0.187). TG increased by about 7 mg/dL in the treatment group but fell by roughly 1 mg/dL in the placebo group (*P* = 0.386). Hs-CRP increased by 0.28 mg/dL in the treatment group but decreased by 0.09 mg/dL in the placebo group (*P* = 0.336).

**Conclusions:**

We found that adding RYR (Lesstat®) to statin medications significantly decreases total cholesterol. However, no significant effect was seen on other lipid profile components or Hs-CRP. Finally, we showed that RYR is safe to add to statins considering liver function (clinicaltrials.gov: NCT05095480).

## Introduction

The prevalence of chronic diseases were estimated about 21% worldwide [1]. Cardiovascular diseases (CVDs) are a leading cause of mortality and morbidity. Atherosclerosis is a chronic inflammatory disease that leads to CVDs such as coronary artery disease (CAD). There are many risk factors for atherosclerosis, the most prominent of which are the male sex, smoking, dyslipidemia, family history, and elevated age [2–6].

Dyslipidemia is one of the most important causes of CVD as it leads to inflammatory processes and deposition of plaques within arteries [7]. Currently, treatment of dyslipidemia includes lifestyle modifications and the use of lipid-lowering medications such as statins, fibrates, and ezetimibe [8]. Lifestyle modifications include dietary changes. Using phytosterols found in herbal foods can improve lipid profile [9]. *Nigella sativa* is one of the herbal medicine that leads to reducing CVD with an anti-inflammation effect [10]. Statins are the first pharmacological choice for treating dyslipidemia in patients at high risk for CVDs [11] [1].

For several reasons, several studies have looked at going beyond statins and benefiting from other agents. Firstly, statins are not usually prescribed for patients with low to moderate risk of CVD or borderline levels of LDL-C; lifestyle modifications are recommended instead. Secondly, statins are not tolerated by all patients and sometimes cause side effects. Finally, alternative agents are associated with certain drawbacks. For example, the recently approved anti-PCSK9 monacolin antibodies are expensive and are not readily available in all countries. Furthermore, ezetimibe agents are inadequate to reach goals in patients with high CVD risk, though they provide a 15–20% reduction in LDL-C levels. Hence, seeking novel agents can open new paths in the lipid-lowering course and reduce the chance of CVD [7, 12].

One novel agent with potential lipid-lowering properties is red yeast rice (RYR), produced from the fermentation of common rice by the mold *Monascus purpureus*. Natives in East Asia have traditionally used this agent for many years, and novel studies have shown that RYR can lower blood lipid levels and reduce CVD risk. The lipid-lowering process is dependent on a substance named monacolin K, which is structurally identical to lovastatin – a type of statin. The mechanism of action of monacolin K is 3-hydroxy-3-methyl-glutaryl-CoA reductase (HMG-CoA reductase) inhibition. HMG-CoA reductase is the main enzyme involved in hepatic cholesterol synthesis [13, 14].

As previous studies have demonstrated, RYR can reduce the CVD risk and is effective in patients with statin intolerance or patients who do not achieve goals after single therapy with statins or ezetimibe. RYR is also known as the most effective compound available on the market for treating hyperlipidemia. It has been claimed that consuming a specific dosage of monacolin K existing in RYR every day leads to an appropriate level of LDL-C reduction. Myalgia is one side effect seen in some patients who consume RYR; these patients also have resistance to low-dose statins. Generally, RYR is a safe and good choice for patients with dyslipidemia but no other risk factors [15–17].

We conducted a new trial by prescribing RYR to dyslipidemia patients already on single statin therapy. We also assessed the effect of RYR on the inflammatory marker. We aimed to observe the effect of RYR in reaching LDL-C targets, keeping in mind possible effects on other lipid profile components and hepatic function.

## Methods

### Study design and study participants

This triple-blind randomized clinical trial involved 92 patients and was performed in 2019. Using the block randomization technique (block size = 2; ratio of 2:2 for drug vs. placebo), 43 patients were randomly allocated into the treatment arm, while 49 patients comprised the control arm. We selected the patients from those referring to the Prof. Kojuri Cardiology Clinic (Niayesh St., Shiraz, Iran, www.kojuriclinic.com, Instagram @Kojuri_clinic). All participants were thoroughly informed about the study process and participated voluntarily after providing informed consent. The study was approved by the Professor kojuri Ethical committee, whith the number of KCEC-99 − 12, and is registered with clinicaltrials.gov (NCT05095480, 27/10/2021), where the study protocol is publically available.

### Inclusion and exclusion criteria

Adults with serum LDL-C levels ≥100 mg/dL under treatment with a statin for at least 12 weeks were eligible for inclusion in this trial. Excluded were individuals with extremely high LDL-C levels (> 200 mg/dL), orlistat hypersensitivity, use of alternative lipid-lowering agents alongside the statin, or a history of hepatic disease. Pregnant or lactating women were also excluded. Patients with other chronic disease such as hypothyroidism were excluded.

### Intervention

Initially, all potential participants were evaluated in terms of serum LDL-C, high-density lipoprotein cholesterol (HDL-C), total cholesterol, triglyceride (TG), and high sensitivity C-reactive protein (hs-CRP) levels. The criteria mentioned above were applied following these tests.

Red yeast rice (RYR) supplements (Lesstat® tablets, Gricar Chemical Srl Co.) were prescribed to the patients in the treatment arm. The tablets included 200 mg of red rice fermented by *Monascus purpureus* (tit. 5% in monacolin K). Among the constituents were 10 mg of monacolin K, 90 mg of chitosan, 3.5 mg of lycopene, 30 mg of ascorbic acid, and 5 mg of tocopherol. Placebo tablets, similar in shape and color, were prescribed to the patients in the control arm. All tablets were packaged identically, with 30 tablets of RYR or placebo being prescribed for daily use to each participant. The tablets were coded by staff external to the research group such that the researchers were kept blinded until after statistical analysis. We instructed the study subjects to consume one tablet per day alongside their routine statin therapy for 30 days and immediately contact us if any problems developed.

### Sample size

We determined the sample size by using the following formule.

$$n=\left[\left( Z\alpha /2+ Z\beta \right)2\times \right\{\left(p1\ \left(1-p1\right)+\left(p2\ \left(1-p2\right)\right)\right\}\Big]/\left(p1-p2\right)2$$

A sample size of 92 patients is sufficient to detect a clinically important difference between two groups with 80% power and a 5% level of significance.

### Data collection

We asked the subjects to attend a follow-up visit after 1 month, at which time the initial blood tests were repeated. The initial and final blood tests were performed via the same standardized methods using the same kits in the same laboratory. At the end of the study period, we also assessed possible adverse hepatic effects of combination (statin and RYR) therapy through serum assays for aspartate transaminase (AST), alanine transaminase (ALT), and total bilirubin.

### Statistical analysis

Statistical analysis was performed using SPSS for Windows ver. 25 (IBM Corp., Armonk, NY**,** USA). To compare the baseline measures across the study groups, the independent t-test and chi-squared test were used where appropriate. To compare initial and final measures, the paired-sample t-test and repeated measure ANOVA were used where applicable. Statistical significance was indicated when *P* < 0.05 in all cases. we used the kolmogorov-smirnov normality test for evaluating the normality of variables (page 04).

## Results

**Fig. 1:**
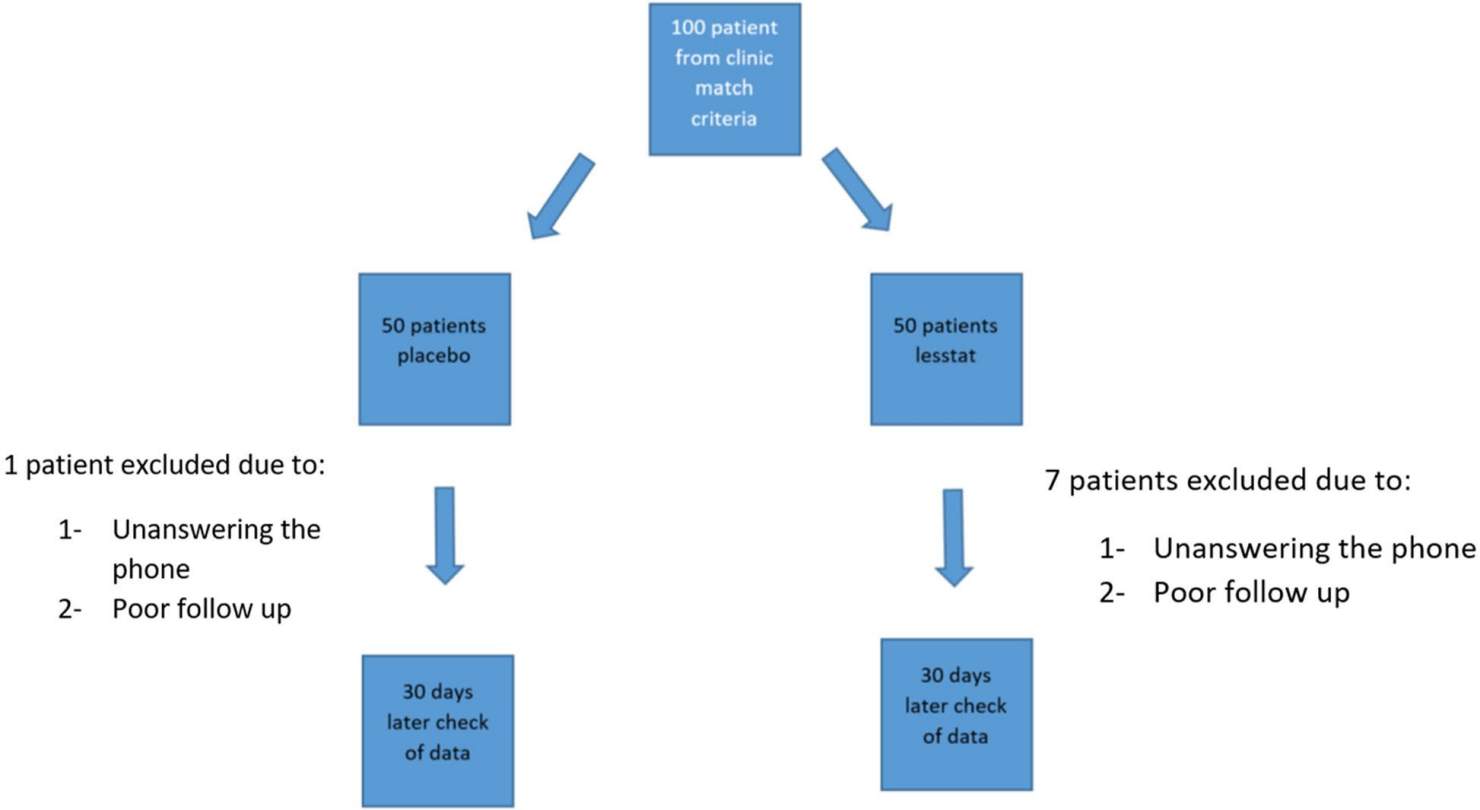
Study protocol flowchart

We included 92 patients (52 women and 50 men) in this study. The mean age of the subjects was about 63 years (minimum age is 53 and maximum is 67). We randomly allocated 43 patients to the medication group, and the other 49 patients comprised the placebo group. At baseline, the groups were comparable in terms of age, gender, the use of different types of statins, and serum levels of TG, HDL-C, and Hs-CRP. However, they differed significantly in total cholesterol and LDL-C levels (Table 1)(Fig. 1).

**Table 1:** Baseline characteristics of patients

	RYR	Placebo	*P*-value
**Age (years), mean ± SD**	63 ± 8	64 ± 9	0.566
Male, n (%)	17	23	0.475
Statin, n (%)			
Atorvastatin 10 mg	3	7	0.671
Atorvastatin 20 mg	16	14	
Atorvastatin 40 mg	4	9	
Rosuvastatin 5 mg	8	6	
Rosuvastatin 10 mg	4	3	
Rosuvastatin 20 mg	5	7	
Rosuvastatin 40 mg	3	3	
TG (mg/dL), mean ± SD	133.6 ± 68.3	117.3 ± 63.6	0.23
Total cholesterol (mg/dL), mean ± SD	164.2 ± 34.4	147.1 ± 25	0.007
LDL cholesterol (mg/dL), mean ± SD	100.9 ± 24.2	87.4 ± 20.8	0.005
HDL cholesterol (mg/dL), mean ± SD	43.7 ± 10.1	42.6 ± 12.1	0.642
Hs-CRP (mg/dL), mean ± SD	1.59 ± 2.3	1.4 ± 2.16	0.78
Daily calories intake (kcal/day)	2340 ± 120	2297 ± 139	0.11

*RYR* Red yeast rice,*SD* Standard deviation, *TG* Triglyceride, *LDL* Low-density lipoprotein, *HDL* High-density lipoprotein, *Hs-CRP* High-sensitivity C-reactive protein

After 1 month, we measured the lipid profile and hs-CRP level again, then calculated the difference between baseline and final measurements (Table 2). Total cholesterol significantly (*P* = 0.019) decreased in the treatment group (− 10.2 mg/dL) compared to the placebo group (− 1.3 mg/dL). HDL-C decreased by about 2.19 mg/dL in the treatment group; however, in the placebo group, HDL-C increased by about 0.53 mg/dL. The difference was not statistically significant (*P* = 0.083). The LDL-C level declined in both placebo (− 5.09) and treatment (− 0.73) groups, but the difference was insignificant (*P* = 0.187). TG increased by about 7 mg/dL in the treatment group but decreased by about 1 mg/dL in the placebo group. However, the difference was not statistically significant (*P* = 0.386).

**Table 2:** Differences between baseline and final measurements

	RYR	Placebo	*P*-value
TG (mg/dL), mean [95% CI]	[− 6, 19]	-1 [−12, 11]	0.386
Total cholesterol (mg/dL), mean [95% CI]	−10.26 [− 16.05, − 4.47]	−1.33 [− 6.27, 3.6]	0.019
LDL cholesterol (mg/dL), mean [95% CI]	−5.09 [−9.66, − 0.52]	− 0.73 [− 5.43, 3.98]	0.187
HDL cholesterol (mg/dL), mean [95% CI])	−2.19 [−4.2, − 0.18]	0.53 [− 1.8, 2.86]	0.083
Hs-CRP (mg/dL), mean [95% CI]	0.28 [−0.24, 0.8]	− 0.09 [− 0.67, 0.48]	0.336

***RYR*** Red yeast rice, *CI* Confidence interval, *TG* Triglyceride, *LDL* Low-density lipoprotein, *HDL* High-density lipoprotein, *Hs-CRP* High-sensitivity C-reactive protein

The Hs-CRP level increased in the treatment group by about 0.28 mg/dL but decreased by about 0.09 mg/dL in the placebo group. Again, the difference lacked statistical significance (*P* = 0.336). We also requested liver function assays at the end of the study, yielding comparable results across the study groups (Table 3).

**Table 3:** Liver function tests of patients in both groups

	RYR	Placebo	*P*-value
Aspartate transaminase (U/L), mean ± SD	23 ± 6	20 ± 3	0.074
Alanine transaminase (U/L), mean ± SD	24 ± 13	23 ± 7	0.714
Total bilirubin (mg/dL), mean ± SD	0.82 ± 0.46	0.7 ± 0.25	0.439

*RYR* Red yeast rice, *SD* Standard deviation

## Discussion

Given the detrimental consequences of dyslipidemia and in light of certain limitations to statin use, this randomized clinical trial (RCT) sought to assess the effects of red yeast rice (RYR) in patients with dyslipidemia already on statin therapy. We found that RYR, alongside statin therapy, can significantly decrease total cholesterol levels without adversely affecting liver enzymes levels (AST, ALT).

Several meta-analyses have confirmed the strong relationship between LDL levels and the risk of cardiovascular disease (CVD) [18]. One meta-analysis by the Cholesterol Treatment Trialists’ (CTT) Collaboration worked on data from 14 RCTs and about 90,000 subjects. The study suggested that as the serum level of LDL-C falls, the risk of CVD decreases accordingly [19]. Another CTT meta-analysis on more than 170,000 patients revealed that each time the LDL concentration drops by one mmol/L, the risk of ischemic stroke, coronary artery disease, and revascularization drops by over one-fifth [20]. Because of the benefits of LDL-C reduction, lipid-lowering agents – especially statins – are highly popular.

Recently, RYR has gained popularity as an alternative LDL-lowering agent with few adverse effects [21]. Several meta-analyses have confirmed the effect of RYR on the reduction of LDL-C. One recent study worked on 20 RCTs and 6663 individuals; it showed that after 2 months to 2 years of therapy, RYR decreased the serum LDL-C level by 1.02 mmol/l (~ 39.4 mg/dl) (with 95% confidence) in comparison with the placebo, indicating considerable efficacy similar to that of low-intensity or low-dose statins (pravastatin 40 mg, simvastatin 10 mg, lovastatin 20 mg). The researchers also confirmed a slight increase in HDL-C and an insignificant decrease in TG [14]. Another study showed that patients receiving RYR experienced significant decrements in serum LDL-C (23.0%) and total cholesterol (15.5%) levels relative to a control group following a sixteen-week treatment period (*P* < 0.001) [22]. The lipid-lowering effect of RYR is believed to be due to the presence of monacolin K, which possesses the same structure as lovastatin [23]. It is thought that RYR limits the rate of hepatic cholesterol production by inhibiting the 3-hydroxy-3-methyl-glutaryl-CoA (HMG-CoA) reductase enzyme [17].

One consideration in the use of novel lipid-lowering agents is hepatotoxicity. A meta-analysis of seven trials [16, 24–29] assessed serum AST levels before and after intervention with RYR. The researchers showed that although the serum AST level were considerably higher in those who received RYR relative to controls, they remained within the normal range (0–40 U/L) [Total WMD = 1.55 (95% CI: 0.26, 2.84) U/L, I2 = 0%, *P* = 0.02, 7 trials (8 comparisons), *n* = 443]. Our study found that after 1 month of treatment, the serum AST level was only slightly higher in the intervention group than the placebo group (*P* = 0.074) and remained within the normal range. In the mentioned trials [16, 24–29], the serum ALT levels was significantly higher in the intervention group compared with the placebo group but again remained within the normal range (0–40 U/L) [Total WMD = 1.47 (95% CI: 0.42, 2.51) U/L, I2 = 0%, *P* = 0.006, 7 trials (8 comparisons), *n* = 443]. At the end of our study, the ALT level was also negligibly higher in the intervention group relative to the placebo group (*P* = 0.714) and remained within the normal range.

Another important aspect related to RYR is that it may improve endothelial function. In one study, 50 coronary heart disease patients randomly received either RYR (1200 mg daily, containing 11.4 mg of monacolin K) or a placebo, and the serum hs-CRP concentration was monitored. After 6 weeks, those receiving RYR experienced reductions in hs-CRP (*P* < 0.001) [30]. However, in our study, changes in hs-CRP levels after 4 weeks of intervention were not significant compared with the placebo (*P* = 0.78).

Although some recent studies only worked on the effect of RYR in isolation on LDL-C, our study investigated the effect of RYR when accompanied by another statin (atorvastatin or rosuvastatin) on both total cholesterol and LDL-C levels. Due to our limited sample size, further large-scale studies seem warranted. Another limitation was the significant differences in baseline total cholesterol and LDL levels between the study groups. Although the study was randomized, this may be due to the small sample size. We also measured the effect of RYR with only a one-month follow-up; future studies should consider an extended period of follow-up.

### Limitations

Although in this randomized trial, we showed that use of RYR is sfae with statin, this need further larger trials to show the effect of RYS on lipid profile.

## Conclusions

Adding RYR to statin medications significantly decreases the serum level of total cholesterol in patients with dyslipidemia. However, no significant effect was seen on other lipid profile components or Hs-CRP. We also showed that adding RYR to statins is safe considering liver function over 1 month.

## Data Availability

The datasets generated and/or analysed during the current study are not publicly available due to patient data privacy, but are available from the corresponding author on reasonable request.

## References

[1] Jana A, Chattopadhyay A (2022). Prevalence and potential determinants of chronic disease among elderly in India: rural-urban perspectives. PLoS One.

[2] Chiha J (2015). Gender differences in the severity and extent of coronary artery disease. Int J Cardiol Heart Vasc.

[3] Villablanca AC, McDonald JM, Rutledge JC (2000). Smoking and cardiovascular disease. Clin Chest Med.

[4] Schaftenaar F (2016). Atherosclerosis: the interplay between lipids and immune cells. Curr Opin Lipidol.

[5] Eaton CB (1996). Family history, and premature coronary heart disease. J Am Board Fam Pract.

[6] Maroszyńska-Dmoch EM, Wożakowska-Kapłon B (2016). Clinical and angiographic characteristics of coronary artery disease in young adults: a single Centre study. Kardiol Pol.

[7] Banach M (2019). The role of red yeast rice (RYR) supplementation in plasma cholesterol control: a review and expert opinion. Atheroscler Suppl.

[8] Booth JN (2016). Healthy lifestyle factors and incident heart disease and mortality in candidates for primary prevention with statin therapy. Int J Cardiol.

[9] Nattagh-Eshtivani E (2022). Biological and pharmacological effects and nutritional impact of phytosterols: a comprehensive review. Phytother Res.

[10] Hadi V (2021). Nigella sativa in controlling type 2 diabetes, cardiovascular, and rheumatoid arthritis diseases: molecular aspects. J Res Med Sci.

[11] Karr S (2017). Epidemiology, and management of hyperlipidemia. Am J Manag Care.

[12] Barter PJ, Rye K-A (2016). New era of lipid-lowering drugs. Pharmacol Rev.

[13] Yang CW, Mousa SA (2012). The effect of red yeast rice (Monascus purpureus) in dyslipidemia and other disorders. Complement Ther Med.

[14] Gerards MC (2015). Traditional Chinese lipid-lowering agent red yeast rice results in significant LDL reduction but safety is uncertain–a systematic review and meta-analysis. Atherosclerosis.

[15] Parra-Virto A (2018). Usefulness of compounds with monacolin K in a case of statins intolerance. Clínica e Investigación en Arteriosclerosis (English Edition).

[16] Becker DJ (2009). Red yeast rice for dyslipidemia in statin-intolerant patients: a randomized trial. Ann Intern Med.

[17] Cicero AF, Fogacci F, Banach M (2019). Red yeast rice for hypercholesterolemia. Methodist DeBakey Cardiovasc J.

[18] Hobbs FR (2016). Is statin-modified reduction in lipids the most important preventive therapy for cardiovascular disease? A pro/con debate. BMC Med.

[19] Unit, E.S (2005). Efficacy and safety of cholesterol-lowering treatment: prospective meta-analysis of data from 90 056 participants in 14 randomised trials of statins. Lancet.

[20] Baigent C, et al. Efficacy and safety of more intensive lowering of LDL cholesterol: a meta-analysis of data from 170 000 participants in 26 randomised trials. Lancet. 2010;376(9753):1670–81. 10.1016/S0140-6736(10)61350-5 PMC2988224 21067804

[21] Fogacci F (2019). Safety of red yeast rice supplementation: a systematic review and meta-analysis of randomized controlled trials. Pharmacol Res.

[22] Bogsrud MP (2010). HypoCol (red yeast rice) lowers plasma cholesterol–a randomized placebo controlled study. Scand Cardiovasc J.

[23] Endo A (1979). Monacolin K, a new hypocholesterolemic agent produced by a Monascus species. J Antibiotics.

[24] Barrat E (2013). Effect on LDL-cholesterol of a large dose of a dietary supplement with plant extracts in subjects with untreated moderate hypercholesterolaemia: a randomised, double-blind, placebo-controlled study. Eur J Nutr.

[25] Lee I-T (2012). Combined extractives of red yeast rice, bitter gourd, chlorella, soy protein, and licorice improve total cholesterol, low-density lipoprotein cholesterol, and triglyceride in subjects with metabolic syndrome. Nutr Res.

[26] Karl M (2012). A multicenter study of nutraceutical drinks for cholesterol (evaluating effectiveness and tolerability). J Clin Lipidol.

[27] Marazzi G (2011). Long-term effects of nutraceuticals (berberine, red yeast rice, policosanol) in elderly hypercholesterolemic patients. Adv Ther.

[28] Yang N-C (2009). Combined nattokinase with red yeast rice but not nattokinase alone has potent effects on blood lipids in human subjects with hyperlipidemia. Asia Pac J Clin Nutr.

[29] Heber D (1999). Cholesterol-lowering effects of a proprietary Chinese red-yeast-rice dietary supplement. Am J Clin Nutr.

[30] Zhao SP (2004). Xuezhikang, an extract of cholestin, protects endothelial function through antiinflammatory and lipid-lowering mechanisms in patients with coronary heart disease. Circulation.

